# Embolic Protection with the TriGuard 3 System in Nonagenarian Patients Undergoing Transcatheter Aortic Valve Replacement for Severe Aortic Stenosis

**DOI:** 10.3390/jcm11072003

**Published:** 2022-04-02

**Authors:** Alexander Lind, Rolf Alexander Jánosi, Matthias Totzeck, Arjang Ruhparwar, Tienush Rassaf, Fadi Al-Rashid

**Affiliations:** 1 Department of Cardiology and Vascular Medicine, West-German Heart and Vascular Center Essen, University of Duisburg-Essen, 45147 Essen, Germany; alexander.janosi@uk-essen.de (R.A.J.); matthias.totzeck@uk-essen.de (M.T.); tienush.rassaf@uk-essen.de (T.R.); fadi.al-rashid@uk-essen.de (F.A.-R.); 2 Department of Heart Surgery, West-German Heart and Vascular Center Essen, University of Duisburg-Essen, 45147 Essen, Germany; arjang.ruhparwar@uk-essen.de

**Keywords:** TAVR, nonagenarians, embolic protection, stroke, interventional devices, percutaneous valve therapy

## Abstract

Background: Transcatheter aortic valve replacement (TAVR) improves the survival and life quality of nonagenarian patients with aortic stenosis. Stroke remains one of the most worrisome complications following TAVR. Cerebral embolic protection devices (CEPDs) may reduce neurological complications after TAVR. This study evaluated the safety and efficacy of CEPDs during TAVR in nonagenarian patients. Methods: Between January 2018 and October 2021, 869 patients underwent transfemoral TAVR (TF-TAVR) at our center. Of these, 51 (5.9%) patients were older than ninety years. In 33 consecutive nonagenarian patients, TF-TAVR was implanted without CEPDs using balloon-expandable valves (BEVs) and self-expandable valves (SEVs). Eighteen consecutive nonagenarians underwent TF-TAVR using a CEPD (CP group). Follow up period was in-hospital or 30 days after the procedure, respectively. Results: Minor access site complications occurred in two patients (3.9%) and were not CEPD-associated. Postinterventional delirium occurred in nine patients (17.6%). Periprocedural minor non-disabling stroke and delirium occurred in ten patients (19.6%). Periprocedural major fatal stroke occurred in two patients in the BEV group (3.9%). Two patients in the BEV group died due to postinterventional pneumonia with sepsis. The mortality rate was 7.8%. The results did not differ between the groups. Conclusions: Age alone is no longer a contraindication for TAVR. CEPD using the Triguard 3 system in nonagenarian TAVR patients was feasible and safe and did not increase access site complications.

## 1. Introduction

With improved survival, the prevalence of valvular heart disease is steadily increasing. In nonagenarians, the most prevalent valvular heart disease, estimated as high as 9.9%, is aortic stenosis [1]. Transcatheter aortic valve replacement (TAVR) continues to expand rapidly as a less invasive option for the treatment of severe aortic stenosis for patients across all risk spectra [2,3], and the current literature is considering TAVR to be the therapy of choice for nonagenarians (Patients > 90 years) with severe aortic stenosis [4].

A substantial proportion of patients submitted to TAVR continues to be affected by procedure-related neurological events, with 30-day clinical stroke rates in the range of 4–7% [5,6,7]. Especially in elder patients, cerebrovascular events (CVEs), such as postinterventional delirium, transient ischemic attack (TIA) or stroke, are devastating adverse events and have been identified as independent predictors of increased mortality and morbidity during follow-up [8]. CVEs have a strong impact on life quality, impairing cognitive function and daily abilities jeopardizing the beneficial effect of TAVR mobilizing nonagenarian patients early and rehabilitating the elder patient back to their usual state [9,10,11].

In order to reduce the risk of CVEs, several cerebral embolic protection devices (CEPDs) have recently been developed. Their use during TAVR showed reduced cerebral lesions assessed by cMRI (cerebral Magnetic Resonance Imaging) in single-center studies, multiple large studies and meta-analyses [12,13,14,15,16,17]. Nonagenarians are commonly excluded from randomized controlled trials, and thus, the evidence for the very elderly is inadequate [18]. Our objective was to describe the feasibility, safety and impact on the outcome of using a transfemoral implantable CEPD, the Triguard 3^TM^ (TG3) deflection device, during transfemoral TAVR in a high-risk cohort of all-comers nonagenarian patients.

## 2. Materials and Methods

### 2.1. Patient Population

Between January 2018 and October 2021, 869 patients underwent TF-TAVR at the West German Heart and Vascular Center [19]. Of these, 51 (5.9%) patients were ninety years and older. In 33 consecutive nonagenarian patients, a TF-TAVR was implanted without a CEPD. Subsequently, eighteen consecutive nonagenarians underwent TF-TAVR using a CEPD independent of anatomical conditions.

This retrospective single-center all-comers observational study was performed in accordance with the Declaration of Helsinki. The study protocol was approved by the ethics committee of the Faculty of Medicine of the University of Duisburg-Essen (No. 16–7080-BO). All parameters were analyzed anonymously. Written informed consent was obtained from each patient following a comprehensive assessment and discussion in the multidisciplinary Heart Valve Team meeting and was deemed best managed with TAVR.

Aortic stenosis (AS) severity was assessed using transthoracic echocardiography (TTE) according to the joint *European Society of Echocardiography* recommendations [20]. Preoperative imaging was performed in all patients using electrocardiogram-gated multidetector contrast computed tomography angiography. Image analysis, including three-dimensional reconstructions, was performed using *3mensio Structural Heart* software version 9.1 (Pie Medical Imaging, Maastricht, The Netherlands). A transfemoral approach was obtained in each case.

The study population was initially divided into two groups comparing CEPD patients with non-CEPD patients documenting no difference concerning neurological events and access site complications (Supplementary Table S1). Only delirium was trending towards significance in the CEPD group. To balance for possible bias because a CEPD was only used in patients receiving BEV, thereafter the study population was divided into three groups: nonagenarian patients receiving TAVR without a CEPD using balloon-expandable valves (BEVs) or self-expandable valves (SEVs) or nonagenarian patients treated with TAVR and CEPDs using only balloon-expandable valves (cerebral protection group (CPG)).

### 2.2. TriGuard 3^TM^ Device

The TG3 (Keystone Heart, Tampa, FL, USA) is a current Conformité Européenne mark-approved device. The TG3 is a temporary, retrievable, single-use and single-sized, self-expanding deflection filter composed of a structural radiopaque nitinol frame and an ultra-thin polymer mesh (nominal pore size 115 × 145 μm) that allows maximal blood flow to the brain covering all three major cerebral vessels while diverting emboli towards the descending aorta. The device is heparin-coated to reduce thrombogenicity and increase lubricity. The full system also includes a delivery subsystem for crimping and loading the device into an 8F sheath [21,22].

### 2.3. TAVR Procedure and Operative Technique

All cases were performed without sedation to facilitate communication between the operator, anesthesiologist, and patient and reduce post-operative delirium [23,24,25]. Transfemoral vascular access and closure of the delivery catheter were obtained percutaneously. A radial artery line was used for invasive blood pressure monitoring, and two peripheral venous access lines were used for drug and volume administration whenever necessary. Central venous access was obtained via the left or right femoral vein to place a pacemaker for right atrium pacing. A left- or right-sided 7F femoral arterial sheath was placed for a cross-over placement of an Amplatz Super Stiff^TM^ Guidewire (Boston Scientific Corporation, Marlborough, MA, USA) to facilitate endovascular repair of the contralateral common femoral artery whenever necessary [25]. The TG3 system was introduced through a contralateral 8F arterial sheath hosting the device and the pigtail catheter under fluoroscopic guidance after performing an aortic angiogram. Additional access for a pigtail catheter is not necessary.

The TG3 was positioned in the proximal aortic arch under fluoroscopic guidance through a contralateral 8F femoral sheath to cover all three major cerebral arteries, namely the innominate, left common carotid, and subclavian arteries and is anchored by the device frame’s circumferential apposition against the aortic arch.

Self-expandable valves (CoreValve Evolut Pro valve (Medtronic Inc., Minneapolis, MN, USA)) or balloon-expandable valves (Sapien S3 Ultra bioprostheses (Edwards Lifesciences, Irvine, CA, USA)) with a current Conformité Européenne mark approval were implanted in cerebral unprotected cases at the discretion of the physician. In all cerebral protected cases, balloon-expandable valves (Sapien S3 Ultra (Edwards Lifesciences, Irvine, CA, USA)) were implanted.

In case of using a Medtronic Evolut Pro 26 mm and 29 mm valve an 18F Medtronic Sentrant Sheath (Medtronic Inc., Minneapolis, MN, USA) was used for vessel access. In the case of the Medtronic Evolut Pro 34 mm, the 16F Medtronic InLine Sheath (Medtronic Inc., Minneapolis, MN, USA) was used. A 14F or 16F Edwards eSheath (Edwards Lifescience Inc., Irvine, CA, USA) guided by a standard 180 cm 0.035 guidewire was inserted femorally according to the instruction for use (IFU). Under fluoroscopic guidance, an Amplatzer Left catheter and a straight tipped wire were used to cross the aortic arch below the TG3 device and cross the aortic valve. A pigtail catheter was then used to exchange a Confida Brecker guidewire (Medtronic, Minneapolis, MN, USA) into the left ventricle. Balloon aortic valvuloplasty was performed at the discretion of the operator prior to implant. Unfractionated heparin was administered during the procedure. The initial heparin dose was 70 U/kg body weight, and the activated clotting time (ACT) was measured last before valvuloplasty or the insertion of the valve. If not >250 s, an additional heparin bolus according to body weight was administered.

The valve system was mounted as described in the IFU of the Edwards valve system. The assembly has been advanced carefully through the aortic arch to keep the TG3 system in place until the deployment position is reached.

When satisfactory positioning was achieved, rapid pacing of the Edwards Sapien valve was initiated, and the valve was deployed. In the case of Evolut Pro implantation, only slow pacing was initiated (100–120 beats per second), and the valve was slowly deployed until a final satisfactory position was achieved. After valve implantation, the valve delivery system was withdrawn into the sheath, and an angiogram was taken to confirm the correct positioning of the valve. A transthoracic echocardiogram was used to assess hemodynamic parameters. The valve delivery system was then removed from the body under fluoroscopic guidance. Finally, the TG3 system was removed, and percutaneous closure of arterial puncture sites was performed using the Perclose Proglide suture-mediated closure system (Abbott Cardiovascular, Plymouth, MN, USA).

### 2.4. Study Definitions

Procedural data, including demographic and outcome data, were entered into a dedicated database. Peri- and postprocedural complications were evaluated according to the Valve Academic Research Consortium 3 (VARC-3) [26] and Bleeding Academic Research Consortium (BARC) definitions [27] (Supplementary Table S2). Cerebrovascular events and post-operative delirium were monitored and classified according to the Valve Academic Research Consortium 3 (VARC-3) criteria.

The primary endpoint was peri- and postprocedural safety and a combined endpoint of postinterventional stroke, TIA or delirium. All suspicious for stroke or TIA were seen and stratified by board-certified neurologists, and patients were treated according to current stroke guidelines [28].

### 2.5. Statistical Analysis

Statistical analyses were performed using SPSS 27.0.1.0 (IBM, Armonk, NY, USA). Continuous variables are expressed as mean and standard deviation (SD), whereas categorical variables are presented as numbers and percentages. For categorical data, the χ^2^ test with post hoc Bonferroni test and analysis of variance (ANOVA) with post hoc Tukey test for continuous data were used to test for statistical significance. For all analyses, *p* values < 0.05 were considered statistically significant.

## 3. Results

Patient population and anatomic data: The patients’ baseline characteristics are listed in Table 1. Our study cohort represents a specific transfemoral TAVR population comprising 51 nonagenarian patients (mean age, 91.1 ± 1.9; 43.1% male) with severe symptomatic AS (mean aortic pressure gradient 44.6 ± 16.8 mmHg) and high operative risk due to age and comorbidities (log. EuroScore 23.7 ± 13.8%, EuroScore II 8.2 ± 8.8%). The pre-procedural calculated aortic valve area was 0.6 ± 0.15 cm^2^. Most of the patients were in New York Heart Association (NYHA) classification III/IV (70.6%). Coronary artery disease was documented in 37 patients (72.5%). Percutaneous coronary intervention (PCI) within 6 months before TAVR was performed in 26 (51%) patients.

The mean ejection fraction was 48.7 ± 12.0%. In total, 31 patients (60.8%) had a history of atrial fibrillation, and previous cerebrovascular events were present in 13 patients (25.5%). Peripheral vascular disease was present in 14 patients (27.5%), and cerebral vascular disease was present in 13 patients (25.5%). A history of diabetes was present in 11 patients (21.6%). Impaired renal function defined as GFR < 60 mL/min/1.73 m^2^ was diagnosed in 32 patients (62.7%), mean GFR was 50.3 mL/min/1.73 m^2^ ± 16.1 mL/min/1.73 m^2^. There was no statistically significant difference between the three subgroups.

Procedural characteristics: Table 2 lists the procedural characteristics of the study population and the three study groups. The procedure was successfully completed in 51 patients (100%). All procedures were performed under conscious sedation (100%). Medtronic Evolut Pro Valves (SEV) were implanted in 19 patients. In 32 patients, an Edwards Sapien S3 Ultra valve (BEV) was implanted. Among them, 18 consecutive patients received BEVs (Edwards Sapien S3 Ultra) using cerebral protection (CPG). Valvuloplasty prior to valve implantation was performed in 25 patients across all three groups (49%). Prior valvuloplasty has been performed more often in the SEV group (*n* = 12, 63.2%, *p* = 0.02) compared to the BEV (*n* = 9, 64.3%) and the CPG (*n* = 4, 22.2%) groups. The procedure time was similar in all groups (BEV 58.9 ± 24.4 min vs. SEV 72.2 ± 30.8 min vs. CPG 58.1 min ± 18.8 min, *p* = 0.26). Fluoroscopy time (BEV 9:17 ± 3:14 min vs. SEV 9:49 ± 4:38 min vs. CPG 10:55 ± 3:20 min, *p* = 0.48) and contrast volume (BEV 167 ± 47.5 mL vs. SEV 187.1 ± 48.6 mL vs. CPG 183.9 mL ± 41.9 mL, *p* = 0.46) did not differ significantly. The pre-interventional stay was longer in the BEV group compared to the SEV and the CEP groups (8.4 ± 4.7 days vs. 5.1 ± 3.1 days vs. 5.4 ± 2.2 days, *p* = 0.02).

Adverse Events: Table 3 lists adverse events of the study population and the three study groups. A new, permanent pacemaker implantation was necessary in eight patients (11.5%) and did not differ across the groups (BEV *n* = 3, 21.4% vs. SEV *n* = 3, 15.8%, vs. CPG *n* = 2, 11.1, *p* = 0.73). Type 1 (minor) VARC-3 bleeding complications occurred in 16 patients (31.4%) and did not differ between the groups (BEV *n* = 3, 21.4% vs. SEV *n* = 8, 42.1% vs. CPG *n* = 5, 27.8%, *p* = 0.41). Type 2 (major) VARC-3 bleeding complications occurred in *n* = 8 patients (15.7%) and did not differ between the groups (BEV *n* = 1, 7.1%, SEV *n* = 5, 26.3%, CPG *n* = 2, 11.1%, *p* = 0.26). Only two (3.9%) minor VARC-3 access site complications occurred in both groups. These complications were similar in the BEV compared to the SEV and the CPG (*n* = 0 vs. *n* = 1, 5.3% vs. *n* = 1, 5.6%, *p* = 0.67). There was no case of coronary obstruction, annular rupture or conversion to open surgery.

Cerebrovascular events: Table 4 lists cerebrovascular adverse events of the study population. Postinterventional delirium was present in nine patients (17.6%). This did not differ across the groups (*n* = 3, 21.4% vs. *n* = 4, 21.1% vs. *n* = 2, 11.1%, *p* = 0.66). Periprocedural minor non-disabling stroke and postinterventional delirium was equally distributed between the groups (BEV *n* = 4, 28.8%, SEV *n* = 4, 21.1%, CPG *n* = 2, 11.1%, *p* = 0.46). Periprocedural major fatal stroke occurred in two patients (3.9%) in the BEV group. There was no significant difference between the groups (BEV *n* = 2, 14.3% vs. 0 in the BEV group and 0 in the CPG, *p* = 0.06).

In-hospital mortality was 7.8% driven by 2 patients in the BEV group after peri-interventional major fatal stroke and 2 patients in the SEV group with postinterventional pneumonia and consecutive fatal septic shock. In the first stroke patient in the BEV group, stroke occurred directly after valve deployment. In the second stroke patient in the BEV group, stroke was detected one hour after the intervention. Therefore, stroke might not be associated with the intervention.

## 4. Discussion

In the present analysis, TAVR in nonagenarian patients using cerebral embolic protection devices appears to be equally feasible and safe compared to TAVR in nonagenarians without a CEPD with regard to strokes and delirium, early procedure-related mortality, device success, and other relevant procedural complications.

Postprocedural stroke may lead to devastating clinical consequences as well as a significant economic impact. Patients consider stroke the most important clinical end point, even above death [29]. Nonagenarian patients may be more likely to have extensive calcification of their aortic valve and aortic arch and accordingly have a high risk of cerebral embolization of calcifications during TAVR [30]. Routine neuroimaging studies reveal that ischemic cerebral infarction caused by showers of cerebral emboli during valve instrumentation and placement affects virtually all patients undergoing TAVR [31]. Due to the improvement in transfemoral aortic valve systems and increased knowledge of implanting physicians, the stroke rate in nonagenarian patient cohorts decreased to 4–7% in recent years [6]. In this very frail patient collective, there is a significant interest in strategies to reduce the risk of stroke after TAVI [7].

Cerebral embolic protection devices have been developed to mitigate stroke risk, but the total number of patients randomized in cerebral protection trials to date is small [32]. One observational study using a variable analytic method included 120,000 patients and found no significant reduction in stroke with the use of cerebral embolic protection [33]. Other studies have been associated with improved early imaging, improved clinical neurological outcomes [12,21,34], or even lower mortality in one recent retrospective administrative-data-based analysis [35]. A recent propensity-matched analysis study showed reduced stroke risk in patients under CEPDs [12]. The positive results must be judged carefully due to non-randomized data from a single center in comparison to a historical control group [36,37,38,39,40]. Other studies demonstrated increased stroke rates [41] compared to similar stroke rates in the other report [42].

Patients included in those studies were younger compared to our collective, raising the question of whether nonagenarian patients benefit more from CEPD.

Although the detected stroke rate in our cohort was comparable to other studies, the age was significantly higher in our cohort at the same time [6]. The pre-dilatation rate was high in our cohort due to the massive calcification of the native aortic valve. In the CPG, the pre-dilatation rate was significantly lower compared to the unprotected groups. This may be due to the individual operator’s decision, optimized computed tomography imaging quality over time, and the associated lack of necessity for sizing valvuloplasty. Pre-dilatation increases the manipulation of the native valve and might contribute to higher stroke rates [34,43,44]. Nevertheless, we found no difference in minor non-disabling or disabling stroke rates in the cerebral protected groups compared to unprotected patients.

Several studies addressed the safety of TAVR in nonagenarian patients with contradictory results. Some studies reported worse in-hospital outcomes concerning mortality in nonagenarian TAVR patients compared to octogenarian patients [41,42]. Other studies reported no difference in mortality in nonagenarian TAVR compared to octogenarian patients [6,45]. This is in line with our study reporting an overall in-hospital mortality rate of 7.8% and no significant difference between the protected group compared to the unprotected groups. Mortality in our cohort was triggered by postinterventional pneumonia and major disabling stroke in the protected group.

Femoral access of the Triguard 3 system might increase access site and bleeding complications. In our cohort, minor and major bleeding complications (VARC-3 Type 1 and Type 2) occurred in 31.4% and 15.7%, respectively, but did not differ between the groups. This is in line with other studies analyzing TAVR in nonagenarian patients without cerebral protection systems [7]. However, our complication rates are higher compared to previous studies analyzing the performance of the Triguard 3 system in younger patients, which reported lower bleeding and access site complication [22]. Other outcomes of nonagenarian patients receiving CEPDs in our cohort, such as the use of a contrast agent, AKI, procedure and fluoroscopy time, and total length of hospital stay, were comparable between the groups. Device positioning was successful in all patients protecting all three aortic arch takeoffs in our cohort. No CEPD dislocation was recognized before the valve deployment step of TAVR. This is a higher success rate compared to other studies, such as the DEFLECT I and II studies, which have shown a lower positioning success rate [22,46,47].

Since 18–27% of new diffusion-weighted images (DWI) in MRI lesions after TAVR are detected in the cerebellum and brainstem, it seems to make sense to protect also the left-sided cerebral arteries being the case with the Triguard 3 deflection device [48,49]. In this context, the recent Clean-TAVI study showed that almost 50% of new DWI lesions are missed with partial coverage using protection systems sparing out the left-sided cerebral arteries [15].

However, while the coverage of the left-sided cerebral arteries is important and the manipulation of any vessels that perfuse the brain, such as the brachiocephalic trunk and left common carotid artery, needs to be avoided to prevent the dislodgement of atherosclerotic material, it is equally important to avoid any unnecessary arch manipulation that could occur with a device that is deployed in the aortic arch.

### Study Limitations

Since our study included a special and rarely investigated patient cohort of nonagenarians, the number of included patients was small (5.9% of the overall cohort). Given recent trial results [36,50], using different valve types and taking very low stroke rates into account a much higher number of patients would be necessary to find a difference between the valve types using a CEPD that reaches any statistical significance.

The present study is a single-center retrospective observational report with potential methodology-inherent bias that is common to this study type. Due to age or distance to the implanting center, most of the patients did not present at the outpatient clinic at three-month or one-year follow-ups, resulting in an inability to report on VARC-3 adverse events beyond the hospital stay or 30 days, respectively.

No neurocognitive function tests were performed, but neurological function was examined by board certified neurologists.

No pre-interventional or postinterventional MRIs were performed to quantify cerebral lesion numbers and volume. In previous studies, MRI lesion volume and numbers did correspond to neurocognition scores but not to prognosis. However, there was no difference in neurocognition between patients treated with and without cerebral protection [51].

## 5. Conclusions

The current study shows that the use of the TriGuard 3 system for cerebral protection during TAVR in nonagenarian patients is feasible and safe and was not associated with an increased adverse event rate. Further randomized studies comparing the use of the Triguard 3 system in a larger nonagenarian cohort with different valve systems are warranted to evaluate whether CPED is superior in this particular patient cohort.

## Figures and Tables

**Table 1:** Baseline characteristics of the study population.

Variables	Overall (*n* = 51)	Unprotected Group (BEV) (*n* = 14)	Unprotected Group (SEV) (*n* = 19)	Cerebral Protection Group (CPG) (*n* = 18)	*p*-Value
Age (years)	91.9 ± 1.9	92.4 ± 2.1	91.6 ± 1.6	91.7 ± 2.1	0.52
Male patients	22 (43.1)	8 (57.1)	6 (31.6)	8 (44.4)	0.34
Body mass index (kg/m^2^)	24.8 ± 3.3	25.4 ± 3.5	24.6 ± 3.8	24.6 ± 2.9	0.76
NYHA III/IV	36 (70.6)	10 (71.4)	13 (68.4)	13 (72.2)	0.97
Coronary artery disease	37 (72.5)	13 (92.9)	14 (73.7)	10 (55.6)	0.06
Prior percutaneous coronary intervention	26 (51)	7 (50)	12 (63.2)	7 (38.9)	0.34
Atrial fibrillation	31 (60.8)	10 (71.4)	10 (52.6)	11 (61.1)	0.55
Prior pacemaker	6 (11.8)	2 (14.3)	1 (5.3)	3 (16.7)	0.53
Previous cerebrovascular event	13 (25.5)	1 (7.1)	4 (21.1)	8 (44.4)	0.05
Peripheral vascular disease	14 (27.5)	3 (21.4)	8 (42.1)	3 (16.7)	0.19
Cerebral vascular disease	13 (25.5)	1 (7.1)	4 (21.1)	8 (44.4)	0.05
Diabetes mellitus	11 (21.6)	5 (35.7)	3 (15.8)	3 (16.7)	0.32
Renal insufficiency (GFR < 60 mL/min/m^2^)	32 (62.7)	9 (64.3)	13 (68.4)	10 (55.6)	0.71
GFR (mL/min/^2^)	50.3 ± 16.1	47.8 ± 16.2	52.0 ± 17.9	50.4 ± 14.6	0.74
Logistic EuroScore (%)	23.7 ± 13.8	27.1 ± 14.4	25.9 ± 13.6	20.4 ± 13.7	0.48
EuroScore II (%)	8.2 ± 8.8	5.2 ± 3.0	7.2 ± 7.8	10.4 ± 11.1	0.33
Echocardiographic variables					
Left ventricular ejection fraction (%)	48.7 ± 12.0	43.7 ± 13.9	51.0 ± 11.7	50.1 ± 10.0	0.19
Aortic Valve Area (cm^2^)	0.62 ± 0.15	0.59 ± 0.14	0.65 ± 0.14	0.63 ± 0.18	0.49
Mean Aortic Pressure Gradient (mmHg)	44.6 ± 16.8	43.4 ± 15.2	47.2 ± 19.3	42.8 ± 15.9	0.74

Data are presented as number (%) or mean ± standard deviation. Chi-Square test (post hoc test: Bonferroni) or single-factor analysis of variance (post hoc test: Tukey). BEV = balloon-expandable valves; SEV = self-expandable valves; CPG = cerebral protection group; NYHA = New York Heart Association; GFR = Glomerular filtration rate; TF-TAVR = transfemoral TAVR.

**Table 2:** Procedural Details.

Variables	Overall (*n* = 51)	Unprotected Group (BEV) (*n* = 14)	Unprotected Group (SEV) (*n* = 19)	Cerebral Protection Group (CPG) (*n* = 18)	*p*-Value
Procedural success	51 (100)	14 (100)	19 (100)	18 (100)	
Procedure Time (min)	63.6 ± 25.7	58.9 ± 24.4	72.2 ± 30.8	58.1 ± 18.8	0.26
Fluoroscopy time (min:sec)	10:04 ± 3:50	9:17 ± 3:14	9:49 ± 4:38	10:55 ± 3:20	0.48
Contrast agent (mL)	180.7 ± 45.8	167.9 ± 47.5	187.1 ± 48.6	183.9 ± 41.9	0.46
Area dosage (cGy × cm^2^)	3023.1 ± 2370.8	2239.4 ± 1698.4	4096.6 ± 3010.7	2499.6 ± 1626.1	0.06
Mean Aortic Pressure Gradient Post-TAVR (mmHg)	9.5 ± 5.0	8.4 ± 3.0	8.5 ± 4.6	11.4 ± 6.3	0.19
Length of stay (days)					
Length of stay pre-interventional (days)	6.1 ± 3.6	8.4 ± 4.7 ^a^	5.1 ± 3.1 ^b^	5.4 ± 2.2 ^b^	0.02
Length of stay postinterventional (days)	7.5 ± 5.6	7.4 ± 3.3	7.9 ± 5.0	7.0 ± 7.6	0.88
Length of stay in IMC/ICU (days)	2.9 ± 3.0	3.9 ± 4.1	3.5 ± 3.1	1.6 ± 1.0	0.06
Total hospital stay (days)	13.5 ± 6.5	15.7 ± 5.0	13.0 ± 6.2	12.4 ± 7.7	0.32
Conscious sedation	50 (98.0)	14 (100)	18 (94.7)	18 (100)	0.42
Prior Valvuloplasty	25 (49.0)	9 (64.3) ^a^	12 (63.2) ^b^	4 (22.2) ^a^	0.02
Valve Size Edwards Sapien 3 Ultra					
20 mm	3 (5.9)	0	0	3 (16.7)	
23 mm	15 (29.4)	8 (57.1)	0	7 (38.9)	
26 mm	13 (25.5)	6 (42.9)	0	7 (38.9)	
29 mm	1 (2.0)	0	0	1 (5.6)	
Valve Size Medtronic Evolut Pro					
26 mm	8 (15.7)	0	8 (42.1)	0	
29 mm	10 (19.6)	0	10 (52.6)	0	
34 mm	1 (2.0)	0	1 (5.3)	0	
Mean Aortic Pressure Gradient post TAVR (mmHg)	9.5 ± 5.0	8.43 ± 3.00	8.5 ± 4.6	11.4 ± 6.3	0.13

Data are presented as number (%) or mean ± standard deviation. Chi-square test (post hoc test: Bonferroni) or single factor analysis of variance (post hoc test: Tukey). Groups with different identification letters ^(a,b)^ differ significantly at the 5% level. BEV = balloon-expandable valves; SEV = self-expandable valves; CPG = cerebral protection group; NYHA = New York Heart Association; ICU = intensive care unit; IMCU = intermediate care unit.

**Table 3:** Adverse Events.

Variables	Overall (*n* = 51)	Unprotected Group (BEV) (*n* = 14)	Unprotected Group (SEV) (*n* = 19)	Cerebral Protection Group (CPG) (*n* = 18)	*p*-Value
New permanent pacemaker	8 (11.5)	3 (21.4)	3 (15.8)	2 (11.1)	0.73
Acute kidney injury					
Stage 1	11 (21.6)	4 (28.6)	2 (10.5)	5 (27.8)	0.34
Stage 2	2 (3.9)	0	0	2 (11.1)	0.15
Stage 3	1 (2.0)	0	1 (5.3)	0	0.42
Annular Rupture	0	0	0	0	
Coronary Obstruction	0	0	0	0	
Conversion to open surgery	0	0	0	0	
VARC-3—Bleeding complications					
Type 1—minor bleeding (BARC Type 2)	16 (31.4)	3 (21.4)	8 (42.1)	5 (27.8)	0.41
Type 2—major bleeding (BARC Type 3a)	8 (15.7)	1 (7.1)	5 (26.3)	2 (11.1)	0.26
VARC-3—Access site complications					
Minor	2 (3.9)	0	1 (5.3)	1 (5.6)	0.67
Major	0	0	0	0	
Death < 30 days	4 (7.8)	2 (14.3)	2 (10.5)	0	0.28

Data are presented as number (%) or mean ± standard deviation. Chi-square test (post hoc test: Bonferroni) or single-factor analysis of variance (post hoc test: Tukey). BEV = balloon-expandable valves; SEV = self-expandable valves; CPG = cerebral protection group; BARC = Bleeding Academic Research Consortium; VARC-3 = Valve Academic Research Consortium.

**Table 4:** Cerebrovascular Events.

Variables	Overall (*n* = 51)	Unprotected Group (BEV) (*n* = 14)	Unprotected Group (SEV) (*n* = 19)	Cerebral Protection Group (CPG) (*n* = 18)	*p*-Value
Neurological complications					
Postinterventional delirium	9 (17.6)	3 (21.4)	4 (21.1)	2 (11.1)	0.66
Periprocedural minor non-disabling stroke, delirium	10 (19.6)	4 (28.6)	4 (21.1)	2 (11.1)	0.46
Periprocedural major disabling stroke	2 (3.9)	2 (14.3)	0	0	0.06

Data are presented as number (%) or mean ± standard deviation. Chi-square test (post hoc test: Bonferroni) or single factor analysis of variance (post hoc test: Tukey). BEV = balloon-expandable valves; SEV = self-expandable valves; CPG = cerebral protection group.

## Data Availability

The data presented in this study are available on request from the corresponding author.

## Supplementary Materials

The following supporting information can be downloaded at: https://www.mdpi.com/article/10.3390/jcm11072003/s1: Table S1: Procedural Details and Adverse Events 2-group Analysis (unprotected group vs. cerebral protection group); Table S2: VARC-3 Criteria.
