# Supplementary material for: Embolic Protection with the TriGuard 3 System in Nonagenarian Patients Undergoing Transcatheter Aortic Valve Replacement for Severe Aortic Stenosis
Source: J Clin Med. 2022 Apr 2;11(7):2003. doi: 10.3390/jcm11072003 (PMC8999484; doi:10.3390/jcm11072003)
Supplement: Supplementary file 1 [file jcm-11-02003-s001.zip › Supplementary.pdf]

Supplemental Material

Table S1: Procedural Details and Adverse Events 2-group Analysis (unprotected group vs. cerebral protection group)

Variables	Overall n=51	Unprotected Group (n=33)	Cerebral Protection Group (n=18)	P-Value
Procedural success	51 (100)	33 (100)	18 (100)	1.0
Procedure Time (min)	64.6 ± 25.7	66.6 ± 28.6	58.1 ± 18.8	0.08
Fluoroscopy time (min:sec)	10:04 ± 3:50	9:36 ± 4:03	10:55 ± 3:20	0.42
Contrast agent (ml)	180.7 ± 45.8	178.9 ± 48.3	182.9 ± 41.9	0.42
Area dosage (cGy*cm^2)	3023.1 ± 2370.8	3308.7 ± 2671.96	2499.6 ± 1626.1	**0.03**
Mean Aortic Pressure Gradient Post-TAVR (mmHg)	9.5 ± 5.0	8.5 ± 3.9	11.4 ± 6.3	0.07
Length of stay (days)				
Length of stay pre-interventional (days)	6.1 ± 3.6	6.5 ± 4.1	5.4 ± 2.2	0.24
Length of stay post-interventional (days)	7.5 ± 5.6	7.7 ± 4.3	7.0 ± 7.6	0.29
Length of stay in IMC / ICU (days)	2.9 ± 3.0	3.7 ± 3.5	1.6 ± 1.0	**0.002**
Total hospital stay (days)	13.6 ± 6.5	14.2 ± 5.8	12.4 ± 7.7	0.06
Conscious sedation	51 (100)			
Prior Valvuloplasty	25 (49.0)	21 (63.6)	4 (22.2)	**0.01**
Annular Rupture	0	0	0	
Coronary Obstruction	0	0	0	
Valve Size Edwards Sapien 3 Ultra				
20mm	3 (5.9)	0	3 (16.7)	0.07
23mm	15 (29.4)	8 (24.2)	7 (38.9)	0.43

26mm	13 (25.5)	6 (18.2)	7 (38.9)	0.19
29mm	1 (2.0)	0	1 (5.6)	0.76
Valve Size Medtronic Evolut Pro				
26mm	8 (15.7)	8 (24.2)	0	0.06
29mm	10 (19.6)	10 (30.3)	0	**0.025**
34mm	1 (2.0)	1 (3.0)	0	1.0
Mean Aortic Pressure Gradient post TAVR (mmHg)	9.5 ± 5.0	8.5 ± 3.9	11.4 ± 6.3	0.08
New permanent pacemaker	8 (11.5)	6 (18.2)	2 (11.1)	0.79
Acute kidney injury				
Stage 1	11 (21.6)	6 (18.2)	5 (27.8)	0.66
Stage 2	2 (3.9)	0	2 (11.1)	0.23
Stage 3	1 (2.0)	1 (3)	0	1.0
Periprocedural stroke	2 (3.9)	2 (6.1)	0	0.76
Postinterventional delirium	8 (15.7)	7 (21.2)	2 (11.1)	0.6
Periprocedural stroke, TIA, Delir	11 (21.6)	10 (30.3)	2 (11.1)	0.17
VARC-3 - Bleeding complications				
Type 1 - minor bleeding (BARC Type 2)	16 (31.4)	11 (33.3)	5 (27.8)	0.93
Type 2 - major bleeding (BARC Type 3a)	8 (15.7)	6 (18.2)	2 (11.1)	0.79
VARC-3 - Access site complications				
Minor	2 (3.9)	1 (3.0)	1 (5.6)	1.0
Major	0	0	0	
Conversion to open surgery	0	0	0	
Death < 30days	4 (7.8)	4 (12.1)	0	0.32

Data are presented as mean ± standard deviation or number (%).
NYHA=New York Heart Association; GFR=Glomerular filtration rate; BARC=Bleeding Academic Research Consortium; VARC-3=Valve Academic Research Consortium; ICU=intensive care unit; IMCU=intermediate care unit

Table S2: VARC-3 Criteria

TIA and Stroke

**All Stroke**	Ischaemic stroke<br>Acute onset of focal neurological signs or symptoms conforming to a focal or multifocal vascular territory within the brain, spinal cord, or retina (NeuroARC Type 1a or 1aH) and fulfilling one of the following criteria:<br>• Signs or symptoms lasting ≥24 h or until death, with pathology or neuroimaging evidence of CNS infarction, or absence of other apparent causes<br>• Symptoms lasting <24 h, with pathology or neuroimaging confirmation of CNS infarction in the corresponding vascular territory<br>Haemorrhagic stroke<br>Acute onset of neurological signs or symptoms due to intracranial bleeding from intracerebral or subarachnoid haemorrhage not due to trauma (NeuroARC Types 1b or 1c)<br>Stroke, not otherwise specified<br>Acute onset of neurological signs or symptoms persisting ≥24 h or until death but without sufficient neuroimaging or pathology evidence to be classified (NeuroARC Type 1d)

	TIA<br>Transient focal neurological signs or symptoms lasting <24 h presumed to be due to focal brain, spinal cord, or retinal ischaemia, but without evidence of acute infarction by neuroimaging or pathology, or with no imaging performed (NeuroARC Type 3a or Type 3aH)
	Delirium without CNS injury<br>Transient non-focal neurological signs or symptoms, typically of variable duration, without evidence of infarction on neuroimaging or pathology, or with no imaging performed (NeuroARC Type 3b)
**Stroke Grading**	
Acute stroke severity	• Mild neurological dysfunction: NIHSS 0-5<br>• Moderate neurological dysfunction: NIHSS 6-14<br>• Severe neurological dysfunction: NIHSS ≥15
Stroke Disability	• Fatal Stroke: death resulting from a stroke<br>• Stroke with disability: mRS score of ≥2 at 90 days and increase of ≥1 from pre-stroke baseline<br>• Stroke without disability: mRS score of 0 (no symptoms) or 1 (able to carry out all usual duties and activities) at 90 days or no increase in mRS category from pre-stroke baseline
Neurological events timing	• Periprocedural: Occurring ≤30 days after the index procedure<br>• Acute: Occurring ≤24 h after the index procedure<br>• Sub-acute: Occurring >24 h and ≤30 days after the index procedure

	• Early: Occurring >30 days and ≤1 year after the index procedure<br>• Late: Occurring >1 year after the index procedure

Bleeding

Type 1	• Overt bleeding that does not require surgical or percutaneous intervention, but does require medical intervention by a health care professional, leading to hospitalization, an increased level of care, or medical evaluation (BARC 2)<br>• Overt bleeding that requires a transfusion of 1 unit of whole blood/red blood cells (BARC 3a)
Type 2	• Overt bleeding that requires a transfusion of 2–4 units of whole blood/red blood cells (BARC 3a)<br>• Overt bleeding associated with a haemoglobin drop of >3 g/L (>1.86 mmol/L) but <5 g/d (<3.1 mmol/L) (BARC 3a)
Type 3	• Overt bleeding in a critical organ, such as intracranial, intraspinal, intraocular, pericardial (associated with haemodynamic compromise/ tamponade and necessitating intervention), or intramuscular with compartment syndrome (BARC 3b, BARC 3c) Overt bleeding causing hypovolemic shock or severe hypotension (systolic blood pressure <90 mmHg lasting >30 min and not responding to volume resuscitation) or requiring vasopressors or surgery (BARC 3b)

	• Overt bleeding requiring reoperation, surgical exploration, or reintervention for the purpose of controlling bleeding (BARC 3b, BARC 4)<br>• Post-thoracotomy chest tube output ≥2 L within a 24 h period (BARC 4)<br>• Overt bleeding requiring a transfusion of ≥5 units of whole blood/red blood cells (BARC 3a)<br>• Overt bleeding associated with a haemoglobin drop ≥5 g/dL (≥3.1 mmol/L) (BARC 3b).
Type 4	Overt bleeding leading to death. Should be classified as:<br>• Probable: Clinical suspicion (BARC 5a)<br>• Definite: Confirmed by autopsy or imaging (BARC 5b)

Acute kidney injury (AKNI classification)

Stage 1	Increase in serum creatinine to 150–199% (1.5–1.99 × increase compared with baseline) OR increase of ≥0.3 mg/dl (≥26.4 mmol/l) OR Urine output <0.5 ml/kg/h for >6 but <12 h
Stage 2	Increase in serum creatinine to 200–299% (2.0–2.99 × increase compared with baseline) OR Urine output <0.5 ml/kg/h for >12 but <24 h
Stage 3	Increase in serum creatinine to ≥300% (>3 × increase compared with baseline) OR serum creatinine of ≥4.0 mg/dl (≥354 mmol/l) with an acute increase of at least 0.5 mg/dl (44 mmol/l) OR Urine output <0.3 ml/kg/h for ≥24 h OR Anuria for ≥12 h

Vascular access site and access-related complications

Major vascular complications	One of the following:<br>• Aortic dissection or aortic rupture<br>• Vascular (arterial or venous) injury (perforation, rupture, dissection, stenosis, ischaemia, arterial or venous thrombosis including pulmonary embolism, arteriovenous fistula, pseudoaneurysm, haematoma, retroperitoneal haematoma, infection) or compartment syndrome resulting in death, VARC type ≥2 bleeding, limb or visceral ischaemia, or irreversible neurologic impairment<br>• Distal embolization (non-cerebral) from a vascular source resulting in death, amputation, limb or visceral ischaemia, or irreversible end-organ damage<br>• Unplanned endovascular or surgical intervention resulting in death, VARC type ≥2 bleeding, limb or visceral ischaemia, or irreversible neurologic impairment<br>• Closure device failure resulting in death, VARC type ≥2 bleeding, limb or visceral ischaemia, or irreversible neurologic impairment
Minor vascular complications	One of the following:<br>• Vascular (arterial or venous) injury (perforation, rupture, dissection, stenosis, ischaemia, arterial or venous thrombosis including pulmonary embolism, arteriovenous fistula, pseudoaneurysm, haematoma, retroperitoneal haematoma, infection) not resulting in death, VARC type ≥2 bleeding, limb or

	visceral ischaemia, or irreversible neurologic impairment n Distal embolization treated with embolectomy and/or thrombectomy, not resulting in death, amputation, limb or visceral ischaemia, or irreversible end-organ damage<br>• Any unplanned endovascular or surgical intervention, ultra-sound guided compression, or thrombin injection, not resulting in death, VARC type ≥2 bleeding, limb or visceral ischaemia, or irreversible neurologic impairment<br>• Closure device failure not resulting in death, VARC type ≥2 bleeding, limb or visceral ischaemia, or irreversible neurologic impairment
Major access-related non-vascular complications	One of the following:<br>• Non-vascular structure, non-cardiac structure§ perforation, injury, or infection resulting in death, VARC type ≥2 bleeding, irreversible nerve injury or requiring unplanned surgery or percutaneous intervention<br>• Non-vascular access site (e.g., trans-apical left ventricular) perforation, injury, or infection resulting in death, VARC type ≥2 bleeding, irreversible nerve injury or requiring unplanned surgery or percutaneous intervention
Minor access-related non-vascular complications	One of the following:<br>• Non-vascular structure, non-cardiac structure perforation, injury, or infection not resulting in death, VARC type ≥2, irreversible

	nerve injury, or requiring unplanned surgery or percutaneous intervention<br>• Non-vascular access site (e.g., trans-apical left ventricular) perforation, injury, or infection not resulting in death, VARC type ≥2 bleeding, irreversible nerve injury or requiring unplanned surgery or percutaneous intervention
